# Mathematical analysis of a cancer model with time-delay in tumor-immune interaction and stimulation processes

**DOI:** 10.1186/s13662-021-03621-4

**Published:** 2021-10-26

**Authors:** Kaushik Dehingia, Hemanta Kumar Sarmah, Yamen Alharbi, Kamyar Hosseini

**Affiliations:** 1 Department of Mathematics, Gauhati University, Guwahati, Assam India; 2 Department of Mathematics, King Abdul Aziz University, Jeddah, Saudi Arabia; 3 Department of Mathematics, Rasht Branch, Islamic Azad University, Rasht, Iran

**Keywords:** 37M05, 37M10, 37N25, 92B05, Tumor-immune model, Delay, Stability, Hopf bifurcation, Numerical simulations

## Abstract

In this study, we discuss a cancer model considering discrete time-delay in tumor-immune interaction and stimulation processes. This study aims to analyze and observe the dynamics of the model along with variation of vital parameters and the delay effect on anti-tumor immune responses. We obtain sufficient conditions for the existence of equilibrium points and their stability. Existence of Hopf bifurcation at co-axial equilibrium is investigated. The stability of bifurcating periodic solutions is discussed, and the time length for which the solutions preserve the stability is estimated. Furthermore, we have derived the conditions for the direction of bifurcating periodic solutions. Theoretically, it was observed that the system undergoes different states if we vary the system’s parameters. Some numerical simulations are presented to verify the obtained mathematical results.

## Introduction

Cancer can be classified as abnormal growth and uncontrolled division of normal cells. The development of cancer is a complex phenomenon. The research community still does not understand the growth law of cancer and the immune system’s response in a tumor’s presence. However, research has proven that the immune system can eliminate tumor cells once they recognize those as malignant. So, in recent years, research on tumor-immune dynamics has gained more interest for the applied mathematicians, biologists, oncologists, and scientists. Mathematical modeling is an intelligent tool to gain insight into any real-world complex system. For example, COVID-19 disease has been creating havoc throughout the world during the last two years. Rezapour *et al.* [1] presented an SEIR epidemic model for the transmission of COVID-19 using the Caputo fractional derivative to find some remedial measures. Based on actual data and fitted in the model, the authors predicted the transmission of COVID-19 for the world in general and Iran in particular. In work [2], Rezapour and Mohammadi studied an AH1N1 influenza model by using the Caputo–Fabrizio fractional-order derivative. They calculated the model results for different fractional order and compared those with the results of the integer-order model. Aydogan *et al.* [3] examined a Caputo–Fabrizio fractional-order mathematical model of Rabies disease with the use of the Laplace Adomian decomposition method. A box model of mumps-induced hearing loss in children using the Caputo–Fabrizio fractional-order derivative that preserves the system’s historical memory was investigated in [4]. The authors also determined the optimal control problem for the proposed model considering treatment as a control parameter to reduce the infected population. By examining the sensitivity of basic reproduction numbers to each of the model parameters, they showed that the basic reproduction number increases with the increase of disease transmission rate and daily birth rate. Also, it reduces with an increase in the recovery rate, normal mortality rate.

The tool of mathematical modeling is widely used by researchers in the field of cancer modeling too. Several significant works [5–9] through mathematical modeling have been done to understand the response of the immune system with tumor. In [5], the authors analyzed a cancer model by considering the interactions between cancer cells, tumor angiogenesis and endothelial cells. Lopez *et al.* [6] estimated the decay rate of tumor cells in the presence of immune response and set a threshold value for which the immune system can eradicate the tumor. Dong *et al.* [7] explored the effect of $\mathrm{CD}4^{+}T$ cells in a tumor-immune system incorporated with adoptive cellular immunotherapy $(\mathit{ACI})$ and suggested that $\mathrm{CD}4^{+}T$ cells play a crucial role in the tumor eradication process under *ACI* therapy. Arlotti *et al.* [8] proposed a bilinear model of integro-differential equations to describe the dynamics of cellular interaction between tumor and immune cells. By introducing two phases, namely, the Inter-phase and M-phase at which cells are generally divided, Awang *et al.* [9] newly described the interactions between tumor cells and immune system. Zeng and Ma [10] analyzed a deterministic tumor-immune model under the Allee effect. This Allee effect disturbed the growth and reproduction of tumor-immune cells. They found a range of Allee threshold values for which the system can be stabilized.

The cytotoxic *T* cells are primarily responsible for tumor suppression and are found in all tissues in the body. Kuznetzov *et al.* [11] described the response of *CTL* cells to the growth of an immunogenic tumor. Quinonez *et al.* [12] examined a mathematical model to show the immune response in the presence of synthetic tumor vaccines to mitigate developing cancer. Li and Li [13] modified the Kuznetsov *et al.* [11] and Galach model [14] to a stochastic one by perturbing environmental noise. They found that the reduced rate of tumors in their model is faster than in the earlier models. Also, their result reveals that environmental noise is favorable for the extinction of tumor cells under immune surveillance, and noise is ineffective when the immune system’s ability is strong enough. De Pillis *et al.* [15] explored the role of natural killer $(\mathit{NK})$ and $\mathrm{CD}8^{+}T$ cells in the immune-tumor interaction mechanism and tumor surveillance through a mathematical model. Another mathematical model was developed by Dritschel *et al.* [16] to investigate the anti-tumor immune response of helper and cytotoxic *T* cells. They concluded that the tumor growth could be reduced if we use treatments like *IL*-2 therapy, adoptive *T* cell therapy which boosts the immune system, and antibody therapy which blocks tumor-induced immuno-suppression. Recently, Pang *et al.* [17] proposed a modified model of [18, 19] to reflect the clinical phenomena of anti-tumor immune responses and observed that the tumor cells show initial exponential growth to the final stable position at zero depending upon the flow rate of mature immune cells.

In many biological complex systems, time delay plays a vital role. The introduction of time delay forces a system to depend not only on the present state but also on the past state. In cancer modeling, the time delay can describe the required time for cell differentiation, cell proliferation, the response of one cell to other cells, etc. Banerjee and Sarkar [20] studied a delay-induced tumor-immune model to control the growth of malignant cells. They varied parameters, analyzed the model, and observed that Hopf bifurcation occurs for delay term as the bifurcation parameter. Rihan *et al.* [21] considered a family of differential models to explore the effects of *ACI* therapy to control tumor burden. In a study of Bi and Xiao [22], they illustrated the bifurcation analysis of a modified version of the Kuznetsov model [11] with the introduction of two-time delays for immune response into the model. Khajanchi [23] and Khajanchi *et al.* [24] analyzed in detail the influence of time delay on the chaotic dynamics of tumor-immune interaction model [25]. In [23, 24], the authors presented that in the presence of delay, the model shows long-term chaotic behavior with Hopf bifurcations. They also estimated the length of the time delay to preserve stability and direction of Hopf bifurcation. Ghosh *et al.* [26] described the interaction between tumor cells and micro-environment immune and host cells with the use of two time delays, one for immune interaction with tumor and the other for immune action on the tumor. Dong *et al.* [27] converted their previously proposed model [7] to a delayed one with the use of two delays, namely the immune activation delay for *ECs* and immune activation delay for *HTCs*. Their results showed that the unstable equilibrium goes to a stable position for the activation delay of *HTCs*. In the work [28], the authors modified the model proposed by Dong *et al.* [7] with the use of one delay term for the activation of *ECs* by *HTCs*. This delay term induced two effects on the model. The first effect is that stability switches to instability, and the second stabilizes tumor-presence equilibrium. Further modification of the model [7] was carried out by Das *et al.* [29] with the use of distributed and discrete-time delay. They observed that uniform activation of helper *T*-cells can help in *ECs* stimulation and tumor control. Considering a reaction-diffusion system, including time delay under the Neumann boundary conditions, Kayan *et al.* [30] modified the model [11] which described tumor-immune competitions. They studied the Hopf bifurcation analysis and found that the effect of diffusion of tumor-immune interaction can significantly change the dynamics of the model.

In this article, we investigate the model proposed by Pang *et al.* [17] introducing delay term for anti-tumor immune responses of matured *T* lymphocytes to destroy tumor cells. In Sect. 2, we formulate our proposed model. A qualitative analysis is done in Sect. 3. In Sect. 4, conditions of Hopf bifurcation of the system are analyzed. Section 5 and Sect. 6 deal with the stability of the limit cycle and direction and stability of Hopf bifurcation. Numerical examples, discussion, and conclusions are carried out in respective Sects. 7 and 8.

## The model

Mathematical models can give a better insight into tumor-immune interaction. In the above literature, we have found that each model is proposed to understand the tumor cells’ mechanism and reduce the tumor burden in the body. *T* lymphocytes are the most important cells in the immune system, capable of killing the tumor cells through kinetic processes. However, it is important to note that the tumor cells can also compete with *T* lymphocytes and make them functionally inactive. Also, tumor cells secrete cytokines, which are responsible for tumor cells proliferation. As a result, eradicating all tumor cells becomes difficult for *T* lymphocytes, and a time delay occurs for the deactivation of tumor cells by *T* lymphocytes. This time delay is regarded as an interaction and stimulation delay of the tumor-immune system. We account for this time delay, the model of Pang *et al.*[17] is thus modified into a delay system as follows:

2.1 $$ \left . \begin{aligned} &\frac{dL_{1}(\tau )}{d\tau } = \mu - \lambda _{1}L_{1}(\tau ) + \alpha _{1} \frac{T(\tau -\Delta )L_{2}(\tau -\Delta )}{\eta + T(\tau -\Delta )}, \\ &\frac{dL_{2}(\tau ))}{d\tau } = \lambda _{1}L_{1}(\tau )- \alpha _{3}L_{2}( \tau ), \\ &\frac{dT(\tau )}{d\tau } = \lambda _{2}T(\tau )-\alpha _{2}T(\tau - \Delta )L_{2}(\tau -\Delta ), \end{aligned} \right \} $$

where $L_{1}(\tau )$, $L_{2}(\tau )$, and $T(\tau )$ are densities of immature *T* lymphocytes, mature *T* lymphocytes, and tumor cells at any time *τ* respectively.

In the first equation of (2.1), the first term *μ* describes the fixed production rate of immature *T* lymphocytes by the body in the absence of tumor cells. The second term $\lambda _{1}L_{1}(\tau )$ is used for describing the transformation rate of immature *T* lymphocytes to mature *T* lymphocytes. The third term $\alpha _{1} \frac{T(\tau -\Delta )L_{2}(\tau -\Delta )}{\eta + T(\tau -\Delta )}$ describes the recruitment term of anti-tumor immune response, where $\alpha _{1}$ is the maximum recruitment rate and *η* is the half-saturation constant. Here, Δ represents the discrete-time delay factor added for interaction and stimulation delay of the tumor-immune system. The second equation describes the dynamics of mature *T* lymphocytes where $\alpha _{3}$ is the inactivation rate of *T* lymphocytes. The third equation designates the rate of change of tumor cells in which tumor cells can grow exponentially in the absence of immune response, where $\lambda _{2}$ is the exponential tumor growth rate. The term $\alpha _{2}T(\tau -\Delta )L_{2}(\tau -\Delta )$ describes the interaction between tumor and mature *T* lymphocytes, where $\alpha _{2}$ is the rate of tumor cells killed by the mature *T* lymphocytes.

For the sake of discussion of model (2.1), we substitute $\mu = \lambda _{0}L_{0}$ and use nondimensional variables and parameters as

$$ (x,y,z) = \biggl(\frac{\alpha _{2}}{\alpha _{1}}\biggl(L_{1}- \frac{\lambda _{0}}{\lambda _{1}}L_{0}\biggr), \frac{\alpha _{2}}{\lambda _{1}}L_{2}, \frac{T}{\eta } \biggr) \quad \mbox{with } t = \lambda _{1} \tau, $$

and

$$ (a_{1}, a_{2}, a_{3}, a_{4}) = \biggl( \frac{\alpha _{1}}{\lambda _{1}}, \frac{\alpha _{3}}{\lambda _{1}}, \frac{\alpha _{2}\lambda _{0}}{\lambda _{1}^{2}}L_{0}, \frac{\lambda _{2}}{\lambda _{1}} \biggr). $$

The normalized model of (2.1) is

2.2 $$ \left . \begin{aligned} &\frac{dx}{dt} = -x + \frac{y(t-\Delta )z(t-\Delta )}{1+ z(t-\Delta )}, \\ &\frac{dy}{dt} = a_{1}x - a_{2}y + a_{3}, \\ &\frac{dz}{dt} = a_{4}z - y(t-\Delta )z(t-\Delta ), \end{aligned} \right \} $$

with initial history:

2.3 $$ x(\theta ) = \phi _{1}(\theta ),\qquad y(\theta ) = \phi _{2}(\theta ),\qquad z( \theta ) = \phi _{3}(\theta ), $$

with $\phi _{i} \geq 0$, $\forall i = 1, 2, 3$ for $\theta \in [-\Delta , 0]$, where $\phi _{i}(\theta ) \in \mathbb{R}^{3}_{+}$ are the continuous functions on $[\Delta , 0)$ that may display jumps at $\theta = 0$.

## Qualitative analysis

### Basic properties

The following proposition establishes the nonnegativity of the solutions of (2.2) with (2.3).

#### Theorem 3.1

*The solution* $(x(t), y(t), z(t))$ *of system* (2.2) *is nonnegative under the nonnegative initial conditions* ($\phi (i)$, $i = 1, 2, 3$) *defined on* $[0, +\infty )$.

#### Proof

System (2.2) can be written as

3.1 $$ \dot{X} = \begin{pmatrix} \dot{x}(t) \\ \dot{y}(t) \\ \dot{z}(t) \end{pmatrix} = \begin{pmatrix} -x + \frac{y(t-\Delta )z(t-\Delta )}{1+ z(t-\Delta )} \\ a_{1}x - a_{2}y + a_{3} \\ a_{4}z - y(t-\Delta )z(t-\Delta ) \end{pmatrix} = \begin{pmatrix} \mathcal{V}_{1}(X) \\ \mathcal{V}_{2}(X) \\ \mathcal{V}_{3}(X) \end{pmatrix} = \mathcal{V}(X), $$

where the function $\mathcal{V}: \mathbb{R}^{3}_{+} \mapsto \mathbb{R}^{3}$ for $\mathcal{V} \in C^{\infty }(\mathbb{R}^{3}_{+})$ is defined in the nonnegative octant $\mathbb{R}^{3}_{+}$. The R.H.S. of system (3.1) is locally Lipschitz, and hence the derivatives are bounded, satisfy the conditions

3.2 $$ \mathcal{V}_{i}(X) |_{X_{i}(t)},\quad X \in \mathbb{R}^{3}_{+} = \mathcal{V}_{i}(0) \geq 0 \ \forall i = 1, 2, 3. $$

According to the lemma by Yang *et al.* [31], every solution of system (2.2) with initial values (2.3), $\phi _{i}(t) \in \mathbb{R}^{3}_{+}$, say, $X(t) = X[t; X(0)]$, $\forall t > 0$, that is, it remains positive throughout the region $\mathbb{R}^{3}_{+}$, $\forall t > 0$. □

System (2.2) without time delay was discussed in [17]. Since inducing time delay does not affect the existence conditions for equilibria of the system, the system has three equilibria with $E_{0}(0, 0, 0)$ as a trivial equilibrium, $E_{1} (0, \frac{a_{3}}{a_{2}}, 0)$ as a tumor-free one, which always exist, and $E_{2} (\frac{a_{2}a_{4}-a_{3}}{a_{1}}, a_{4}, \frac{a_{2}a_{4}-a_{3}}{a_{3}+a_{1}a_{4}-a_{2}a_{4}} )$ as a co-axial equilibrium, which exists for $\max \{0, (a_{2}-a_{1}a_{4})\}< a_{3} < a_{2}a_{4}$.

- For $a_{3} > a_{2}a_{4}$, the tumor-free equilibrium $E_{1} (0, \frac{a_{3}}{a_{2}}, 0)$ exists.
- There exists only one equilibrium $E_{1}$ for $a_{2} > a_{1}$ and $0 < a_{3} < (a_{2} - a_{1})a_{4}$.
- For $a_{2} < a_{1}$ and $0 < a_{3} < a_{2}a_{4}$, two equilibria $E_{1}$ and $E_{2}$ exist.
- For $a_{2} > a_{1}$ and $(a_{2}-a_{1})a_{4} < a_{3} < a_{2}a_{4}$, two equilibria $E_{1}$ and $E_{2}$ exist.

### Local stability

In order to check the local stability at each of the equilibrium points, we calculate the following Jacobian matrix for the system (2.2):

3.3 $$ \mathcal{J}_{E} = \begin{pmatrix} -1 & \frac{z}{1+z}e^{-\lambda \Delta } & \frac{y}{(1+z)^{2}}e^{- \lambda \Delta } \\ a_{1} & -a_{2} & 0 \\ 0 & -ze^{-\lambda \Delta } & a_{4}-ye^{-\lambda \Delta } \end{pmatrix} . $$

I. The “no living cell” fixed point $E_{0}(0, 0, 0)$ is stable if $a_{4} < 0$. As corresponding to $E_{0}$, the eigenvalues of the matrix (3.3) are $\lambda _{0, 1} = -1$ (<0), $\lambda _{0, 2} = -a_{2}$ (<0), and $\lambda _{0, 3} = a_{4}$.
II. The eigenvalues of the matrix (3.3) are $\lambda _{1, 1} = -1$, $\lambda _{1, 2} = -a_{2}$, and $\lambda _{1, 3} = -\frac{a_{3}-a_{2}a_{4}}{a_{2}}$ corresponding to the tumor-free fixed point $E_{1} (0, \frac{a_{3}}{a_{2}}, 0)$. So, $E_{1}$ is stable if $a_{3} > a_{2}a_{4}$, otherwise unstable. For the case of $a_{2} > a_{1}$ and $0 < a_{3} < (a_{2} - a_{1})a_{4}$, the fixed point $E_{1}$ is a saddle-focus point with two negative eigenvalues and one positive eigenvalue.
III. Now, we will investigate the dynamical behavior of the system (2.2) around the co-axial fixed point $E_{2} (\hat{x}=\frac{a_{2}a_{4}-a_{3}}{a_{1}}, \hat{y}= a_{4}, \hat{z} = \frac{a_{2}a_{4}-a_{3}}{a_{3}+a_{1}a_{4}-a_{2}a_{4}} )$ under the influence of discrete time lag Δ for both the cases of existence of $E_{2}$. In this case, the eigenvalues of matrix (3.3) can be found from the following equation:
  3.4 $$ \begin{aligned} & \lambda ^{3} + \bigl(1+a_{2} - a_{4}+\hat{y}e^{-\lambda \Delta } \bigr) \lambda ^{2} \\ &\quad {}+ \biggl\{ a_{2}-a_{4}-a_{2}a_{4}+ \biggl(a_{2}\hat{y}+\hat{y}- \frac{a_{1}\hat{z}}{1+\hat{z}} \biggr)e^{-\lambda \Delta } \biggr\} \lambda \\ &\quad {}+ \biggl\{ -a_{2}a_{4}+ \biggl(\frac{a_{1}a_{4}\hat{z}}{1+\hat{z}}+a_{2} \hat{y} \biggr)e^{-\lambda \Delta } \biggr\} = 0. \end{aligned} $$
  For the case of no time lag ($\Delta = 0$), equation (3.4) becomes
  3.5 $$ \begin{aligned} & \lambda ^{3} + (1+a_{2} - a_{4}+\hat{y})\lambda ^{2} \\ &\quad {} + \biggl(a_{2}-a_{4}-a_{2}a_{4}+a_{2} \hat{y}+\hat{y}- \frac{a_{1}\hat{z}}{1+\hat{z}} \biggr)\lambda \\ &\quad {} + \biggl(-a_{2}a_{4}+\frac{a_{1}a_{4}\hat{z}}{1+\hat{z}}+a_{2} \hat{y} \biggr)= 0. \end{aligned} $$
  By the use of Routh–Hurwitz criterion to (3.5), $E_{2}$ is asymptotically stable if the following conditions are satisfied:
  3.6 $$ \left . \begin{aligned} & 1+a_{2}-a_{4}+ \hat{y} > 0 \quad \implies\quad 1+a_{2} > 0, \\ & \biggl(-a_{2}a_{4}+\frac{a_{1}a_{4}\hat{z}}{1+\hat{z}}+a_{2} \hat{y} \biggr) > 0 \quad \implies\quad a_{1}a_{4} > 0, \\ & (1+a_{2}-a_{4}+\hat{y}) \biggl(a_{2}-a_{4}-a_{2}a_{4}+a_{2} \hat{y}+\hat{y}- \frac{a_{1}\hat{z}}{1+\hat{z}} \biggr) - \frac{a_{1}a_{4}\hat{z}}{1+\hat{z}} > 0 \\ & \quad \implies \quad (1+a_{2}) \biggl(a_{2}-\frac{a_{1}\hat{z}}{1+\hat{z}} \biggr) - \frac{a_{1}a_{4}\hat{z}}{1+\hat{z}} > 0 \\ &\quad \implies\quad (1+a_{2}) \biggl(a_{2}-\frac{a_{1}\hat{z}}{1+\hat{z}} \biggr)> \frac{a_{1}a_{4}\hat{z}}{1+\hat{z}}. \end{aligned} \right \} $$
  Now, we shall analyze the dynamical behavior of the system (2.2) with time lag $\Delta \neq 0$. For this, we assume that there exists a purely imaginary root for (3.4). Hence, we substitute $\lambda = im (m > 0)$ into (3.4) and, separating the real and imaginary parts, we have
  3.7 $$ \left . \begin{aligned} &(1+a_{2}-a_{4})m^{2}-(-a_{2}a_{4}) = \biggl( \frac{a_{1}a_{4}\hat{z}}{1+\hat{z}}+a_{2}\hat{y} - \hat{y} m^{2} \biggr) \cos (m\Delta ) \\ &\hphantom{(1+a_{2}-a_{4})m^{2}-(-a_{2}a_{4}) ={}} {} + \biggl(a_{2}\hat{y}+\hat{y}-\frac{a_{1}\hat{z}}{1+\hat{z}} \biggr)m\sin (m \Delta ), \\ \text{and}\\ &m^{3} - (a_{2}-a_{2}a_{4}-a_{4})m = \biggl(a_{2}\hat{y}+\hat{y}- \frac{a_{1}\hat{z}}{1+\hat{z}} \biggr)m\cos (m\Delta ) \\ &\hphantom{m^{3} - (a_{2}-a_{2}a_{4}-a_{4})m ={}} {} - \biggl(\frac{a_{1}a_{4}\hat{z}}{1+\hat{z}}+a_{2}\hat{y} - \hat{y} m^{2} \biggr) \sin (m\Delta ). \end{aligned} \right \} $$
  Using the method of cross-multiplication, we solve both the equations of (3.7) and get
  3.8 $$ \tan (m\Delta ) = \frac{c - d}{e + f}, $$
  where
  $$ \begin{aligned} &c = \biggl(a_{2}\hat{y}+\hat{y}- \frac{a_{1}\hat{z}}{1+\hat{z}} \biggr)m \bigl\{ (1+a_{2}-a_{4})m^{2}-(-a_{2}a_{4}) \bigr\} , \\ &d = \bigl\{ m^{3} - (a_{2}-a_{2}a_{4}-a_{4})m \bigr\} \biggl\{ \frac{a_{1}a_{4}\hat{z}}{1+\hat{z}}+a_{2}\hat{y} - \hat{y} m^{2} \biggr\} , \\ &e = \bigl\{ (1+a_{2}-a_{4})m^{2}-(-a_{2}a_{4}) \bigr\} \biggl\{ \frac{a_{1}a_{4}\hat{z}}{1+\hat{z}}+a_{2}\hat{y} - \hat{y} m^{2} \biggr\} , \\ &f = \biggl(a_{2}\hat{y}+\hat{y}-\frac{a_{1}\hat{z}}{1+\hat{z}} \biggr)m \bigl\{ m^{3} - (a_{2}-a_{2}a_{4}-a_{4})m \bigr\} . \end{aligned} $$
  By squaring and adding both sides of both the equations of (3.7), we get
  3.9 $$ m^{6} + p_{0}m^{4} + p_{1}m^{2}+p_{2} = 0, $$
  where
  3.10 $$ \left . \begin{aligned} &p_{0} = (1+a_{2}-a_{4})^{2} - 2(a_{2}-a_{2}a_{4}-a_{4}) - \hat{y}^{2}, \\ &p_{1} = (a_{2}-a_{2}a_{4}-a_{4})^{2}-2(1+a_{2}-a_{4}) (-a_{2}a_{4}) \\ &\hphantom{p_{1} ={}}{} +2\hat{y} \biggl(\frac{a_{1}a_{4}\hat{z}}{1+\hat{z}}+a_{2}\hat{y} \biggr) - \biggl(a_{2} \hat{y}+\hat{y}-\frac{a_{1}\hat{z}}{1+\hat{z}} \biggr)^{2}, \\ &p_{2} = a_{2}^{2}a_{4}^{2} - \biggl\{ \frac{a_{1}a_{4}\hat{z}}{1+\hat{z}}+a_{2}\hat{y} \biggr\} ^{2}. \end{aligned} \right \} $$
  The equation (3.9) will have a positive root if
  $$ p_{0} = (1+a_{2}-a_{4})^{2} - 2(a_{2}-a_{2}a_{4}-a_{4}) - \hat{y}^{2} > 0, $$
  and
  $$ p_{2} = a_{2}^{2}a_{4}^{2} - \biggl\{ \frac{a_{1}a_{4}\hat{z}}{1+\hat{z}}+a_{2}\hat{y} \biggr\} ^{2} < 0. $$
  Suppose that $m_{0}$ is a unique non-negative root of the equation (3.9) such that (3.4) has a pair of purely imaginary roots of the form $\pm im_{0}$. Then from the equation (3.8) the time lag $\Delta _{k}$ corresponding to $m_{0}$ is
  3.11 $$ \Delta _{k} = \frac{1}{m_{0}}\arctan \biggl[ \frac{c_{0} - d_{0}}{e_{0} + f_{0}} \biggr] + \frac{2k\pi }{m_{0}}, \quad k = 0, 1, 2, 3,\ldots, $$
  where
  $$ \begin{aligned} &c_{0} = \biggl(a_{2} \hat{y}+\hat{y}-\frac{a_{1}\hat{z}}{1+\hat{z}} \biggr)m_{0} \bigl\{ (1+a_{2}-a_{4})m_{0}^{2}-(-a_{2}a_{4}) \bigr\} , \\ &d_{0} = \bigl\{ m_{0}^{3} - (a_{2}-a_{2}a_{4}-a_{4})m_{0} \bigr\} \biggl\{ \frac{a_{1}a_{4}\hat{z}}{1+\hat{z}}+a_{2}\hat{y} - \hat{y} m_{0}^{2} \biggr\} , \\ &e_{0} = \bigl\{ (1+a_{2}-a_{4})m_{0}^{2}-(-a_{2}a_{4}) \bigr\} \biggl\{ \frac{a_{1}a_{4}\hat{z}}{1+\hat{z}}+a_{2}\hat{y} - \hat{y} m_{0}^{2} \biggr\} , \\ &f_{0} = \biggl(a_{2}\hat{y}+\hat{y}- \frac{a_{1}\hat{z}}{1+\hat{z}} \biggr)m_{0} \bigl\{ m_{0}^{3} - (a_{2}-a_{2}a_{4}-a_{4})m_{0} \bigr\} . \end{aligned} $$
  Therefore, the co-axial equilibrium $E_{2}$ is locally asymptotically stable under the conditions (3.6) for $\Delta _{k} = 0$. So, this point will also remain stable for $\Delta _{k} < \Delta _{0}$, where $\Delta _{k} = \Delta ^{*}$ at $k = 0$ by Butler’s lemma [24]. This suggest that for $\Delta _{k} > \Delta _{0}$ the co-axial equilibrium $E_{2}$ is unstable. This implies that the tumor cells can proliferate faster if the interaction time delay crosses a given critical value and the system loses its stability at $E_{2}$.

The rest of the work which is discussed in Sect. 4, Sect. 5, and Sect. 6 is inspired and followed by the previous works [20, 23, 24, 32, 33].

## Analysis of Hopf bifurcation

As the equation (3.4) has a complex roots of the form $\lambda = im_{0}$, it implies that the system (2.2) may undergo a Hopf bifurcation at $\Delta = \Delta _{k}$ and around the equilibrium $E_{2}$. Here, we establish a condition for which the system (2.2) undergoes a Hopf bifurcation by using Lemma 1 [17]. For this, first we need to verify the transversality condition $\frac{d(\operatorname{Re}\lambda )}{d\Delta }|_{\Delta =\Delta _{k}} > 0$.

Differentiating (3.4) with respect to Δ gives

4.1 $$ \begin{aligned} & \biggl[ \bigl\{ 3\lambda ^{2} + 2\lambda (1+a_{2}-a_{4})+(a_{2}-a_{2}a_{4}-a_{2}) \bigr\} \\ &\qquad {}+ e^{-\lambda \Delta _{k}} \biggl\{ 2\lambda \hat{y}+a_{2}\hat{y}+\hat{y}- \frac{a_{1}\hat{z}}{1+\hat{z}} \biggr\} \\ &\qquad {} -\Delta e^{-\lambda \Delta _{k}} \biggl\{ \hat{y}\lambda ^{2}+ \biggl(a_{2} \hat{y}+\hat{y}-\frac{a_{1}\hat{z}}{1+\hat{z}} \biggr)\lambda + \frac{a_{1}a_{4}\hat{z}}{1+\hat{z}}+a_{2}\hat{y} \biggr\} \biggr] \frac{d\lambda }{d\Delta _{k}} \\ &\quad = \lambda e^{-\lambda \Delta _{k}} \biggl\{ \hat{y}\lambda ^{2}+ \biggl(a_{2} \hat{y}+\hat{y}-\frac{a_{1}\hat{z}}{1+\hat{z}} \biggr)\lambda + \frac{a_{1}a_{4}\hat{z}}{1+\hat{z}}+a_{2}\hat{y} \biggr\} . \end{aligned} $$

Simplifying equation (4.1), we have

4.2 $$ \begin{aligned} \biggl(\frac{d\lambda }{d\Delta _{k}} \biggr)^{-1} & = \frac{2\lambda ^{3}+(1+a_{2}-a_{4})\lambda ^{2}+a_{2}a_{4}}{-\lambda ^{2}\{\lambda ^{3}+(1+a_{2}-a_{4})\lambda ^{2}+(a_{2}-a_{2}a_{4}-a_{4})\lambda -a_{2}a_{4}\}} \\ &\quad {} + \frac{\hat{y}\lambda ^{2}-(\frac{a_{1}a_{4}\hat{z}}{1+\hat{z}}+a_{2}\hat{y})}{\lambda ^{2}\{\hat{y}\lambda ^{2}+(a_{2}\hat{y}+\hat{y}-\frac{a_{1}\hat{z}}{1+\hat{z}})\lambda +\frac{a_{1}a_{4}\hat{z}}{1+\hat{z}}+a_{2}\hat{y}\}} \\ &\quad {} - \frac{\Delta _{k}}{\lambda }. \end{aligned} $$

Therefore, with the increase of the value of $\Delta _{k}$ the direction of motion of *λ* is given by

4.3 $$\begin{aligned}& \begin{aligned} \Pi & = \operatorname{sign} \biggl\{ \operatorname{Re} \biggl(\frac{d\lambda }{d\Delta _{k}} \biggr)^{-1} \biggr\} _{\lambda = im_{0}} \\ & = \operatorname{sign} \biggl[\operatorname{Re} \biggl( \frac{2\lambda ^{3}+(1+a_{2}-a_{4})\lambda ^{2}+a_{2}a_{4}}{-\lambda ^{2}\{\lambda ^{3}+(1+a_{2}-a_{4})\lambda ^{2}+(a_{2}-a_{2}a_{4}-a_{4})\lambda -a_{2}a_{4}\}} \\ &\quad {} + \frac{\hat{y}\lambda ^{2}-(\frac{a_{1}a_{4}\hat{z}}{1+\hat{z}}+a_{2}\hat{y})}{\lambda ^{2}\{\hat{y}\lambda ^{2}+(a_{2}\hat{y}+\hat{y}-\frac{a_{1}\hat{z}}{1+\hat{z}})\lambda +\frac{a_{1}a_{4}\hat{z}}{1+\hat{z}}+a_{2}\hat{y}\}} - \frac{\Delta _{k}}{\lambda } \biggr) \biggr]_{\lambda = im_{0}}, \end{aligned} \\& \Pi =\frac{1}{m^{2}_{0}} \\& \hphantom{\Pi ={}}{}\times \operatorname{sign} \biggl[ \frac{2m^{6}_{0}+m^{4}_{0}\{(1+a_{2}-a_{4})^{2}-2(a_{2}-a_{2}a_{4}-a_{2})-\hat{y}^{2}\} + \{(\frac{a_{1}a_{4}\hat{z}}{1+\hat{z}}+a_{2}\hat{y})^{2}-a_{2}^{2}a_{4}^{2}\}}{({a_{2}\hat{y}+\hat{y}-\frac{a_{1}\hat{z}}{1+\hat{z}})^{2}m^{2}_{0} +(\frac{a_{1}a_{4}\hat{z}}{1+\hat{z}}+a_{2}\hat{y}-\hat{y}m_{0}^{2})}^{2}} \biggr]. \end{aligned}$$

For $\frac{d(\operatorname{Re}\lambda )}{d\Delta }|_{\Delta =\Delta _{k}} > 0$, from the equation (4.3) we must have

4.4 $$ \left . \begin{aligned} & \bigl\{ (1+a_{2}-a_{4})^{2}-2(a_{2}-a_{2}a_{4}-a_{2})- \hat{y}^{2} \bigr\} > 0,\quad \text{and} \\ & \biggl\{ \biggl(\frac{a_{1}a_{4}\hat{z}}{1+\hat{z}}+a_{2}\hat{y} \biggr)^{2}-a_{2}^{2}a_{4}^{2} \biggr\} > 0. \end{aligned} \right \} $$

### Theorem 4.1

*The co*-*axial equilibrium* $E_{2}$ *is*

(i) *asymptotically stable if* Δ ∈ $[0,\Delta _{k})$,
(ii) *unstable if* $\Delta > \Delta _{k}$,
(iii) *Hopf bifurcation occurs around* $E_{2}$ *if* $\Delta = \Delta _{k}$.

## Stability of limit cycle: length of time lag estimation

In this section, we investigate the stability of bifurcating periodic solutions and estimate the length of time lag preserving the stability of period-1 limit cycle. Consider model (2.2) and the space of all continuous real-valued functions defined on $[-\Delta , +\infty )$, which satisfies the initial history (2.3) on the interval $[-\Delta , 0)$. First, we linearize model (2.2) around the co-axial equilibrium point $E_{2} (\hat{x}=\frac{a_{2}a_{4}-a_{3}}{a_{1}}, \hat{y}=a_{4}, \hat{z}=\frac{a_{2}a_{4}-a_{3}}{a_{3}+a_{1}a_{4}-a_{2}a_{4}} )$, which gives us

5.1 $$ \left . \begin{aligned} &\dot{x} = -x + \frac{\hat{z}}{1+\hat{z}} y(t - \Delta ) + \frac{\hat{y}}{(1 + \hat{z})^{2}}z(t-\Delta ), \\ &\dot{y} = a_{1}x - a_{2}y, \\ &\dot{z} = -\hat{z} y(t-\Delta ) + a_{4}z - \hat{y} z(t-\Delta ). \end{aligned} \right \} $$

Using Laplace transformation into (5.1), we have

5.2 $$ \left . \begin{aligned} &(\omega + 1) \mathcal{L}_{x}(\omega ) = \frac{\hat{z}}{1+\hat{z}}e^{- \omega \Delta } \mathcal{L}_{y}(\omega ) + \frac{\hat{z}}{1+\hat{z}}e^{- \omega \Delta } \mathcal{U}_{y}(\omega ) \\ &\hphantom{(\omega + 1) \mathcal{L}_{x}(\omega ) ={}}{} +\frac{\hat{y}}{1+\hat{z}}e^{-\omega \Delta }\mathcal{L}_{z}(\omega ) + \frac{\hat{y}}{1+\hat{z}}e^{-\omega \Delta }\mathcal{U}_{z}(\omega ) + \bar{x}(0), \\ &(\omega + a_{2})\mathcal{L}_{y}(\omega ) = a_{1}\mathcal{L}_{x}( \omega ) + \bar{y}(0), \\ &\bigl(\omega - a_{4}+\hat{y}e^{-\omega \Delta } \bigr) \mathcal{L}_{z}(\omega ) = -\hat{z}e^{-\omega \Delta } \mathcal{L}_{y}(\omega )-\hat{z}e^{-\omega \Delta } \mathcal{U}_{y}(\omega ) \\ &\hphantom{\bigl(\omega - a_{4}+\hat{y}e^{-\omega \Delta } \bigr) \mathcal{L}_{z}(\omega ) ={}}{} - \hat{y}e^{-\omega \Delta }\mathcal{K}_{z}(\omega ) + \bar{z}(0), \end{aligned} \right \} $$

where $\mathcal{U}_{y}(\omega ) = \int _{-\Delta }^{0}e^{-\omega \Delta } y(t)\,dt$, $\mathcal{U}_{z}(\omega ) = \int _{-\Delta }^{0}e^{-\omega \Delta } z(t)\,dt$ and $\mathcal{L}_{x}(\omega )$, $\mathcal{L}_{y}(\omega )$, and $\mathcal{L}_{z}(\omega )$ are the Laplace transformations of $x(t)$, $y(t)$, and $z(t)$ respectively.

Now, by using the theory provided by Freedman *et al.* [34] and the classical Nyquist criteria, the equilibrium point $E_{2}$ is asymptotically stable if, for the equation

5.3 $$ \begin{aligned} P(\omega ) = {}& \omega ^{3} + \bigl(1+a_{2} - a_{4}+ \hat{y}e^{-\omega \Delta } \bigr)\omega ^{2} + \biggl\{ a_{2}-a_{4}-a_{2}a_{4} \\ & {}+\biggl(a_{2}\hat{y}+\hat{y}-\frac{a_{1}\hat{z}}{1+\hat{z}} \biggr)e^{-\omega \Delta } \biggr\} \omega + \biggl\{ -a_{2}a_{4}+ \biggl( \frac{a_{1}a_{4}\hat{z}}{1+\hat{z}}+a_{2}\hat{y} \biggr)e^{-\omega \Delta } \biggr\} , \end{aligned} $$

the following conditions hold:

5.4 $$\begin{aligned}& \operatorname{Re} P(i\zeta _{0}) = 0, \end{aligned}$$

5.5 $$\begin{aligned}& \operatorname{Im} P(i\zeta _{0}) = 0, \end{aligned}$$

where $\zeta _{0}$ is the minimal nonnegative root of (5.4) and (5.5).

From (5.4),

5.6 $$ \begin{aligned} (1+a_{2}-a_{4}) \zeta _{0}^{2} & = -a_{2}a_{4} + \biggl( \frac{a_{1}a_{4}\hat{z}}{1+\hat{z}}+a_{2}\hat{y} \biggr)\cos (\zeta _{0} \Delta ) - \hat{y}\zeta _{0}^{2}\cos ( \zeta _{0}\Delta ) \\ &\quad {} + \biggl(a_{2}\hat{y}+\hat{y}-\frac{a_{1}\hat{z}}{1+\hat{z}} \biggr)\zeta _{0} \sin (\zeta _{0}\Delta ). \end{aligned} $$

Using the inequalities $\lvert \cos (\zeta _{0}\Delta ) \rvert \leq 1$ and $\lvert \sin (\zeta _{0}\Delta ) \rvert \leq 1$, we get

5.7 $$ \begin{aligned} \bigl\lvert (1+a_{2}-a_{4}) \bigr\rvert \zeta _{0}^{2} & \leq \lvert a_{2}a_{4} \rvert + \biggl\lvert \biggl( \frac{a_{1}a_{4}\hat{z}}{1+\hat{z}}+a_{2}\hat{y} \biggr) \biggr\rvert + \lvert \hat{y} \rvert \zeta _{0}^{2} \\ &\quad {} + \biggl\lvert \biggl(a_{2}\hat{y}+\hat{y}-\frac{a_{1}\hat{z}}{1+\hat{z}} \biggr) \biggr\rvert \zeta _{0}. \end{aligned} $$

From (5.7) we have

5.8 $$ \zeta _{+} \leq\frac{\lvert (a_{2}\hat{y}+\hat{y}-\frac{a_{1}\hat{z}}{1+\hat{z}})\rvert + \sqrt{ (a_{2}\hat{y}+\hat{y}-\frac{a_{1}\hat{z}}{1+\hat{z}})^{2} + 4\{\lvert (1+a_{2}-a_{4}) \rvert - \lvert \hat{y} \rvert \}\{\lvert a_{2}a_{4} \rvert + \lvert (\frac{a_{1}a_{4}\hat{z}}{1+\hat{z}}+a_{2}\hat{y} ) \rvert \}}}{2\{\lvert (1+a_{2}-a_{4}) \rvert - \lvert \hat{y} \rvert \}}. $$

Hence, $\zeta _{0} \leq \zeta _{+}$.

From equation (5.5)

5.9 $$ \left . \begin{aligned} \zeta _{0}^{2} & < (a_{2}-a_{2}a_{4}-a_{4})+ \biggl(a_{2}\hat{y}+\hat{y}- \frac{a_{1}\hat{z}}{1+\hat{z}} \biggr)\cos (\zeta _{0}\Delta ) \\ &\quad {} - \frac{ (\frac{a_{1}a_{4}\hat{z}}{1+\hat{z}}+a_{2}\hat{y} )\sin (\zeta _{0}\Delta )}{\zeta _{0}}+ \hat{y}\zeta _{0}\sin (\zeta _{0}\Delta ). \end{aligned} \right \} $$

Using (5.6), equation (5.9) becomes

5.10 $$ \left . \begin{aligned} &\biggl\{ \biggl( \frac{a_{1}a_{4}\hat{z}}{1+\hat{z}}+a_{2}\hat{y} \biggr) - \hat{y}\zeta _{0}^{2}-(1+a_{2}-a_{4}) \biggl(a_{2}\hat{y}+\hat{y}- \frac{a_{1}\hat{z}}{1+\hat{z}} \biggr) \biggr\} \bigl[\cos (\zeta _{0}\Delta )-1 \bigr] \\ &\qquad {}+ \biggl[ \biggl\{ a_{2}\hat{y}+\hat{y}-\frac{a_{1}\hat{z}}{1+\hat{z}} (1+a_{2}-a_{4})\hat{y} \biggr\} \zeta _{0} + \frac{(1+a_{2}-a_{4}) (\frac{a_{1}a_{4}\hat{z}}{1+\hat{z}}+a_{2}\hat{y} )}{\zeta _{0}} \biggr]\sin ( \zeta _{0}\Delta ) \\ &\quad < (1+a_{2}-a_{4}) (a_{2}-a_{2}a_{4}-a_{4})-(-a_{2}a_{4}) \\ &\qquad {} +(1+a_{2}-a_{4})\biggl(a_{2}\hat{y}+\hat{y}-\frac{a_{1}\hat{z}}{1+\hat{z}} \biggr) - \biggl( \frac{a_{1}a_{4}\hat{z}}{1+\hat{z}}+a_{2}\hat{y} \biggr) + \hat{y}\zeta _{0}^{2}. \end{aligned} \right \} $$

Using the inequality $\zeta _{0}^{2} < (a_{2}-a_{2}a_{4}-a_{4}+a_{2}\hat{y}+\hat{y}- \frac{a_{1}\hat{z}}{1+\hat{z}})$ for $\Delta = 0$, the above equation has the form

5.11 $$ \left . \begin{aligned} &\biggl\{ \biggl( \frac{a_{1}a_{4}\hat{z}}{1+\hat{z}}+a_{2}\hat{y} \biggr) - \hat{y}\zeta _{0}^{2}-(1+a_{2}-a_{4}) \biggl(a_{2}\hat{y}+\hat{y}- \frac{a_{1}\hat{z}}{1+\hat{z}} \biggr) \biggr\} \bigl[\cos (\zeta _{0}\Delta )-1 \bigr] \\ &\qquad {}+ \biggl[ \biggl\{ a_{2}\hat{y}+\hat{y}-\frac{a_{1}\hat{z}}{1+\hat{z}} (1+a_{2}-a_{4})\hat{y} \biggr\} \zeta _{0} + \frac{(1+a_{2}-a_{4}) (\frac{a_{1}a_{4}\hat{z}}{1+\hat{z}}+a_{2}\hat{y} )}{\zeta _{0}} \biggr]\sin ( \zeta _{0}\Delta ) \\ &\quad < (1+a_{2}-a_{4}+\hat{y}) \biggl(a_{2}-a_{2}a_{4}-a_{4}+a_{2} \hat{y}+ \hat{y}-\frac{a_{1}\hat{z}}{1+\hat{z}} \biggr) \\ &\qquad {}- \biggl\{ -a_{2}a_{4}+\frac{a_{1}a_{4}\hat{z}}{1+\hat{z}}+a_{2} \hat{y} \biggr\} . \end{aligned} \right \} $$

Now the first term and the second term of the L.H.S. of (5.11) can be written respectively as

$$ \left . \begin{aligned} &\biggl\{ \biggl(\frac{a_{1}a_{4}\hat{z}}{1+\hat{z}}+a_{2} \hat{y} \biggr) - \hat{y}\zeta _{0}^{2}-(1+a_{2}-a_{4}) \biggl(a_{2}\hat{y}+\hat{y}- \frac{a_{1}\hat{z}}{1+\hat{z}} \biggr) \biggr\} \bigl[\cos (\zeta _{0}\Delta )-1 \bigr] \\ &\quad = 2 \biggl[\hat{y}\zeta _{0}^{2}+(1+a_{2}-a_{4}) \biggl(a_{2}\hat{y}+\hat{y}- \frac{a_{1}\hat{z}}{1+\hat{z}} \biggr)- \biggl( \frac{a_{1}a_{4}\hat{z}}{1+\hat{z}}+a_{2}\hat{y} \biggr) \biggr]\sin ^{2} \biggl( \frac{\zeta _{0}\Delta }{2} \biggr) \\ &\quad \leq \frac{1}{2}\zeta _{+}^{2} \biggl\lvert \biggl\{ \hat{y}\zeta _{0}^{2}+(1+a_{2}-a_{4}) \biggl(a_{2} \hat{y}+\hat{y}-\frac{a_{1}\hat{z}}{1+\hat{z}} \biggr)- \biggl( \frac{a_{1}a_{4}\hat{z}}{1+\hat{z}}+a_{2}\hat{y} \biggr) \biggr\} \biggr\rvert \Delta ^{2}, \end{aligned} \right \} $$

and

$$ \left . \begin{aligned} &\biggl[ \biggl\{ a_{2}\hat{y}+ \hat{y}-\frac{a_{1}\hat{z}}{1+\hat{z}} (1+a_{2}-a_{4}) \hat{y} \biggr\} \zeta _{0} + \frac{(1+a_{2}-a_{4}) (\frac{a_{1}a_{4}\hat{z}}{1+\hat{z}}+a_{2}\hat{y} )}{\zeta _{0}} \biggr]\sin ( \zeta _{0}\Delta ) \\ &\quad \leq \biggl[ \biggl\lvert \biggl\{ a_{2}\hat{y}+\hat{y}- \frac{a_{1}\hat{z}}{1+\hat{z}} (1+a_{2}-a_{4})\hat{y} \biggr\} \biggr\rvert \zeta _{+}^{2} + \bigl\lvert (1+a_{2}-a_{4}) \bigr\rvert \biggl\lvert \biggl( \frac{a_{1}a_{4}\hat{z}}{1+\hat{z}}+a_{2}\hat{y} \biggr) \biggr\rvert \biggr] \Delta _{M}. \end{aligned} \right \} $$

Therefore from (5.11)

5.12 $$ \chi _{1}\Delta ^{2} + \chi _{2}\Delta \leq \chi _{3}, $$

where

$$ \left . \begin{aligned} & \chi _{1} = \frac{1}{2}\zeta _{+}^{2} \biggl\lvert \biggl\{ \hat{y}\zeta _{0}^{2}+(1+a_{2}-a_{4}) \biggl(a_{2} \hat{y}+\hat{y}-\frac{a_{1}\hat{z}}{1+\hat{z}} \biggr)- \biggl( \frac{a_{1}a_{4}\hat{z}}{1+\hat{z}}+a_{2}\hat{y} \biggr) \biggr\} \biggr\rvert , \\ &\chi _{2} = \biggl[ \biggl\lvert \biggl\{ a_{2}\hat{y}+ \hat{y}- \frac{a_{1}\hat{z}}{1+\hat{z}} (1+a_{2}-a_{4})\hat{y} \biggr\} \biggr\rvert \zeta _{+}^{2} + \bigl\lvert (1+a_{2}-a_{4}) \bigr\rvert \biggl\lvert \biggl( \frac{a_{1}a_{4}\hat{z}}{1+\hat{z}}+a_{2}\hat{y} \biggr) \biggr\rvert \biggr], \\ &\chi _{3} = (1+a_{2}-a_{4}+\hat{y}) \biggl(a_{2}-a_{2}a_{4}-a_{4}+a_{2} \hat{y}+\hat{y}-\frac{a_{1}\hat{z}}{1+\hat{z}} \biggr)- \biggl\{ -a_{2}a_{4}+ \frac{a_{1}a_{4}\hat{z}}{1+\hat{z}}+a_{2}\hat{y} \biggr\} . \end{aligned} \right \} $$

Now, equation (5.12) gives

5.13 $$ \Delta _{+} = \frac{1}{2\chi _{1}} \bigl[-\chi _{2} + \sqrt{\chi _{2}^{2}+4 \chi _{1}\chi _{3}} \bigr] \quad \text{for } 0 \leq \Delta \leq \Delta _{+}. $$

Therefore, the system (2.2) preserves the stability around the equilibrium $E_{2}$ for the maximum length of the time lag $\Delta _{+}$ with period-1 limit cycle.

## Direction and stability of Hopf bifurcation

In the previous sections, we have discussed the conditions for which the periodic solutions of the system (2.2) bifurcate from co-axial equilibrium $E_{2}$ at the critical values of $\Delta _{k}$ via the Hopf bifurcation. In this section, we will analyze the direction, stability, and periods of the periodic solutions of the system (2.2) using normal theory and the center manifold theorem developed by Hassard *et al.* [35].

Let $u_{1}(t) = x(t) - \bar{x}$, $u_{2}(t) = y(t)-\bar{y}$, $u_{3}(t) = z(t) - \bar{z}$, $x(t) = u_{1}(\Delta t)$, $y(t) = u_{2}(\Delta t)$, $z(t) = u_{3}(\Delta t)$, and $\Delta = \Delta _{0} + \mu $, where $\Delta _{0}$ is defined by (3.11), and $\mu \in \mathbb{R}$. The system (2.2) can be written as a functional differential equation in $\mathbb{C} = \mathbb{C}([-1,0], \mathbb{R}^{3})$ as

6.1 $$ X^{\prime }= L_{\mu } (X_{t}) + f(\mu , X_{t}), $$

where $X(t) = (x(t), y(t), z(t))^{T} \in {\mathbb{R}^{3}}$, and $L_{\mu }: \mathbb{C} \rightarrow {\mathbb{R}^{3}}$, $f: \mathbb{R} \times \mathbb{C} \rightarrow {\mathbb{R}^{3}}$ are given, respectively, as follows: for $\Phi (t) = (\Phi _{1}(t), \Phi _{2}(t), \Phi _{3}(t))^{T} \in \mathbb{C}([-\Delta , 0], \mathbb{R}^{3})$, we define

6.2 $$ L_{\mu }(\Phi ) = D_{1}\Phi (0) + D_{2}\Phi (-1), $$

and

6.3 $$ f(\mu , \Phi ) = (\Delta _{0}+ \mu )M, $$

where

6.4 $$\begin{aligned}& D_{1} = (\Delta _{0} + \mu ) \begin{pmatrix} -1 & 0 & 0 \\ a_{1} & -a_{2} & 0 \\ 0 & 0 & a_{4} \end{pmatrix} , \qquad D_{2} = (\Delta _{0} + \mu ) \begin{pmatrix} 0 & \frac{\bar{z}}{1+\bar{z}} & \frac{\bar{y}}{(1+\bar{z})^{2}} \\ 0 & 0 & 0 \\ 0 & -\bar{z} & -\bar{y} \end{pmatrix} , \end{aligned}$$

6.5 $$\begin{aligned}& M = \begin{pmatrix} \Phi _{2}(-1)\Phi _{3}(-1) - \Phi _{2}(-1)\Phi _{3}^{2}(-1) + \text{HOT} \\ 0 \\ -\Phi _{2}(-1)\Phi _{3}(-1) \end{pmatrix} , \end{aligned}$$

HOT → higher order terms.

By the Riesz representation theorem, there exists a matrix function $\eta (\theta ,\mu )$ of bounded variation for $\theta \in [-1, 0]$ such that

6.6 $$ L_{\mu }(\Phi ) = \int _{-1}^{0} d\eta (\theta , \mu )\Phi (\theta ) \quad \text{for } \Phi \in \mathbb{C}. $$

For the Dirac delta function *δ*, choose

6.7 $$ \eta (\theta , \mu ) = D_{1}\delta (\theta ) + D_{2}\delta (\theta +1), $$

and for $\Phi \in {\mathbb{C}}^{1}([-1,0],\mathbb{R}^{3})$, define

6.8 $$ A(\mu )\Phi (\theta ) = \textstyle\begin{cases} \frac{d\Phi (\theta )}{d\theta }, & \theta \in [-1,0), \\ \int _{-1}^{0} d\eta (s,\mu )\Phi (s), & \theta = 0, \end{cases} $$

and

6.9 $$ R(\mu )\Phi (\theta ) = \textstyle\begin{cases} 0, & \theta \in [-1,0), \\ f(\mu ,\theta ), & \theta = 0. \end{cases} $$

Hence, system (6.1) is equivalent to the operator equation

6.10 $$ X^{\prime }= A({\mu })X_{t} + R(\mu )X_{t}, $$

where $X_{t}(\theta ) = X(t+\theta )$ for $\theta \in [-1,0)$.

For $\Psi \in {\mathbb{C}^{1}}([-1,0), {(\mathbb{R}^{3})}^{*})$, define

6.11 $$ A^{*}\Psi (s) = \textstyle\begin{cases} -\frac{d\Psi (s)}{ds}, & s \in (0,1], \\ \int _{-1}^{0} d\eta ^{T}(t, 0)\Psi (-t), & s = 0, \end{cases} $$

and the bilinear inner product

6.12 $$ \bigl\langle {\Psi (s), \Phi (\theta )} \bigr\rangle = \bar{ \Psi }(0)\Phi (0) - \int _{\theta = -1}^{0} \int _{\xi = 0}^{\theta }\bar{\Psi }(\xi - \theta )\,d\eta (\theta )\Phi (\xi )\,d\xi , $$

where $\eta (\theta ) = \eta (\theta , 0)$. Then $A(0)$ and $A^{*}$ are adjoint operators. We already assume that $\pm \iota \omega _{0}\Delta _{k}$ are the eigenvalues of $A(0)$. Hence, the eigenvalues of $A^{*}$ are $\mp \iota \omega _{0}\Delta _{k}$. We need to compute the eigenvectors of $A(0)$ and $A^{*}$ corresponding to the eigenvalues $\iota \omega _{0}\Delta _{k}$ and $-\iota \omega _{0}\Delta _{k}$ respectively.

Suppose that $v(\theta ) = (1, v_{1}, v_{2})^{T}e^{\iota \omega _{0}\Delta _{k} \theta }$ is the eigenvector of $A(0)$ corresponding to $\iota \omega _{0}\Delta _{k}$. Then $A(0)v(0) = \iota \omega _{0}\Delta _{k}v(0)$. It follows from the definition of $A(0)$ and (6.3), (6.6) and (6.7) that

$$ \Delta _{k} \begin{pmatrix} -1 & 0 & 0 \\ a_{1} & -a_{2} & 0 \\ 0 & 0 & a_{4} \end{pmatrix} v(0) + \Delta _{k} \begin{pmatrix} 0 & \frac{\bar{z}}{1+\bar{z}} & \frac{\bar{y}}{(1+\bar{z})^{2}} \\ 0 & 0 & 0 \\ 0 & -\bar{z} & -\bar{y} \end{pmatrix} v(-1) = \iota \omega _{0}\Delta _{k}v(0). $$

Then, for $v(-1) = v(0)e^{-\iota \omega _{0}\Delta _{k}}$, we obtain

$$ v_{1} = \frac{a_{1}}{a_{2}+\iota \omega _{0}} ; \qquad v_{2} = - \frac{a_{1}\bar{z}e^{-\iota \omega _{0}\Delta _{k}}}{(a_{2}+\iota \omega _{0}) (\iota \omega _{0}+\bar{y}e^{-\iota \omega _{0}\Delta _{k}})}. $$

In a similar manner, we can obtain the eigenvector $v^{*}(s) = D(1, v_{1}^{*}, v_{2}^{*})^{T}e^{-\iota \omega _{0} \Delta _{k}s}$ of $A^{*}$ corresponding to $-\iota \omega _{0}\Delta _{k}$, where

$$ v_{1}^{*} = \frac{1-\iota \omega _{0}}{a_{1}} ; \qquad v_{2}^{*} = - \frac{\bar{y}}{(1+\bar{y})^{2}(\iota \omega _{0}- \bar{y}e^{-\iota \omega _{0}\Delta _{k}}) e^{-\iota \omega _{0}\Delta _{k}}}. $$

In order to guarantee $\langle {v^{*}(s), v(\theta )}\rangle = 1$, we need to determine the expression of *D*

$$ \begin{aligned} &\bigl\langle {v^{*}(s), v(\theta )} \bigr\rangle \\ &\quad = \bar{D} \bigl(1, \bar{v_{1}}^{*}, \bar{v_{2}}^{*} \bigr) (1, v_{1}, v_{2})^{T} \\ &\qquad {} - \int _{\theta = -1}^{0} \int _{\xi = 0}^{\theta } \bar{D} \bigl(1, \bar{v_{1}}^{*}, \bar{v_{2}}^{*} \bigr)e^{-\iota \omega _{0}\Delta _{k}( \xi - \theta )}\,d\eta (\theta ) (1, v_{1}, v_{2})^{T}e^{\iota \omega _{0} \Delta _{k}\xi } \,d\xi \\ &\quad = \bar{D} \biggl\{ \bigl(1 + \bar{v_{1}}^{*}v_{1} + \bar{v_{2}}^{*}v_{2} \bigr) - \int _{\theta = -1} ^{0} \bigl(1, \bar{v_{1}}^{*}, \bar{v_{2}}^{*} \bigr)\theta e^{ \iota \omega _{0}\Delta _{k}\theta }\,d\eta ( \theta ) (1, v_{1}, v_{2})^{T} \biggr\} \\ &\quad = \bar{D} \biggl\{ \bigl(1 + \bar{v_{1}}^{*}v_{1} + \bar{v_{2}}^{*}v_{2} \bigr) + \biggl\{ \frac{\bar{z}}{1+\bar{z}}v_{1}+\frac{\bar{y}}{(1+\bar{z})^{2}}v_{2}- \bar{z}\bar{v_{2}}^{*}v_{1}-\bar{y} \bar{v_{2}}^{*}v_{2} \biggr\} \Delta _{k}e^{- \iota \omega _{0}\Delta _{k}} \biggr\} . \end{aligned} $$

Therefore we can choose *D* as

$$ \bar{D} = \biggl[ \frac{1}{(1 + \bar{v_{1}}^{*}v_{1} + \bar{v_{2}}^{*}v_{2}) + \{\frac{\bar{z}}{1+\bar{z}}v_{1}+\frac{\bar{y}}{(1+\bar{z})^{2}}v_{2}-\bar{z}\bar{v_{2}}^{*}v_{1}-\bar{y}\bar{v_{2}}^{*}v_{2} \}\Delta _{k}e^{-\iota \omega _{0}\Delta _{k}}} \biggr]. $$

On the other hand, due to adjoint property, we can write $\langle {\Psi , A\Phi }\rangle = \langle {A^{*}\Psi , \Phi }\rangle $.

We have

$$ \begin{aligned} -\iota \omega _{0}\Delta _{k} \bigl\langle {v^{*},\bar{v}} \bigr\rangle & = \bigl\langle {v^{*}, A\bar{v}} \bigr\rangle = \bigl\langle {A^{*}v^{*}, \bar{v}} \bigr\rangle \\ & = \bigl\langle {-\iota \omega _{0}\Delta _{k}v^{*}, \bar{v}} \bigr\rangle \\ & = \iota \omega _{0}\Delta _{k} \bigl\langle {v^{*}, \bar{v}} \bigr\rangle . \end{aligned} $$

Therefore, $\langle {v^{*}, \bar{v}}\rangle = 0$.

Now, we will compute the coordinates describing the center manifold $C_{0}$ at $\mu = 0$. Let $X_{t}$ be the solution of (6.10) when $\mu = 0$. We define

6.13 $$\begin{aligned}& Z(t) = \bigl\langle {v^{*}, X_{t}} \bigr\rangle , \\& W(t,\theta ) = X_{t} - z(t)v(\theta )-\bar{z}(t) \bar{v}(\theta ) = X_{t}( \theta )-2\operatorname{Re} \bigl\{ z(t)v( \theta ) \bigr\} . \end{aligned}$$

On the center manifold $C_{0}$ we have $W(t,\theta ) = W(z(t),\bar{z}(t), \theta )$, where

6.14 $$ W(z,\bar{z},\theta ) = W_{20}(\theta ) \frac{z^{2}}{2}+W_{11}(\theta )z \bar{z} + W_{02}( \theta )\frac{\bar{z}^{2}}{2}+W_{30}(\theta ) \frac{z^{3}}{6}+ \cdots, $$

where *z* and *z̄* are the local coordinates for the center manifold $C_{0}$ in the direction of $\bar{v}^{*}$ and $v^{*}$. Note that *W* is real if $x_{t}$ is real. Here, we are only interested in real solutions. From (6.13), we have

$$ \begin{aligned} \bigl\langle {v^{*}, W} \bigr\rangle & = \bigl\langle {v^{*}, X_{t}-zv-\bar{z}\bar{v}} \bigr\rangle \\ & = \bigl\langle {v^{*},X_{t}} \bigr\rangle -z \bigl\langle {v^{*},v} \bigr\rangle -z \bigl\langle {v^{*}, \bar{v}} \bigr\rangle \\ & = z - z \\ & = 0. \end{aligned} $$

For the solution $x_{t} \in C_{0}$ in (6.10), since $\mu = 0$, hence

$$ \begin{aligned} \dot{z}(t) &= \bigl\langle {v^{*}, \dot{X}_{t}} \bigr\rangle = \bigl\langle {v^{*},A(0)X_{t}+R(0)X_{t}} \bigr\rangle \\ & = \bigl\langle {A^{*}(0)v^{*},X_{t}} \bigr\rangle + \bar{v}^{*}(0)f(0,X_{t}) \\ & = \bigl\langle {-\iota \omega _{0}\Delta _{k}v^{*}, X_{t}} \bigr\rangle + \bar{v}^{*}(0)f_{0}(z, \bar{z}) \\ & = \iota \omega _{0}\Delta _{k}z + \bar{v}^{*}(0)f_{0}(z,\bar{z}) \\ & = \iota \omega _{0}\Delta _{k}z(t)+g(z,\bar{z}), \end{aligned} $$

where

6.15 $$ \begin{aligned} g(z,\bar{z}) & = \bar{v}^{*}(0)f_{0}(z,\bar{z}) \\ & = g_{20}\frac{z^{2}}{2} + g_{11}z\bar{z}+ g_{02} \frac{\bar{z}^{2}}{2}+g_{21}\frac{z^{2}\bar{z}}{2} + \cdots. \end{aligned} $$

From (6.13) and (6.14), it follows that

$$ X_{t} = W(z,\bar{z},\theta ) + zv+ \bar{z}\bar{v}. $$

Thus,

$$ X_{t} = \begin{bmatrix} X_{1t}(\theta ) \\ X_{2t}(\theta ) \\ X_{3t}(\theta ) \end{bmatrix} = \begin{bmatrix} W^{(1)}(z,\bar{z},\theta ) \\ W^{(2)}(z,\bar{z},\theta ) \\ W^{(3)}(z,\bar{z},\theta ) \end{bmatrix} + z \begin{bmatrix} 1 \\ v_{1} \\ v_{2} \end{bmatrix} e^{\iota \omega _{0}\Delta _{k}\theta } + \bar{z} \begin{bmatrix} 1 \\ \bar{v}_{1} \\ \bar{v}_{2} \end{bmatrix} e^{-\iota \omega _{0}\Delta _{k}\theta }, $$

where

$$ \left . \begin{aligned} &X_{1t}(\theta ) = ze^{\iota \omega _{0}\Delta _{k}\theta } + \bar{z}e^{- \iota \omega _{0}\Delta _{k}\theta } + W_{20}^{1}( \theta ) \frac{z^{2}}{2} + W_{11}^{1}(\theta )z \bar{z}+W_{02}^{1}(\theta ) \frac{\bar{z}^{2}}{2} + \cdots, \\ &X_{2t}(\theta ) = v_{1}ze^{\iota \omega _{0}\Delta _{k}\theta } + \bar{v_{1}}\bar{z}e^{-\iota \omega _{0}\Delta _{k}\theta } + W_{20}^{2}( \theta )\frac{z^{2}}{2} + W_{11}^{2}(\theta )z \bar{z}+W_{02}^{2}( \theta )\frac{\bar{z}^{2}}{2} + \cdots, \\ &X_{3t}(\theta ) = v_{2}ze^{\iota \omega _{0}\Delta _{k}\theta } + \bar{v_{2}}\bar{z}e^{-\iota \omega _{0}\Delta _{k}\theta } + W_{20}^{3}( \theta )\frac{z^{2}}{2} + W_{11}^{3}(\theta )z \bar{z}+W_{02}^{3}( \theta )\frac{\bar{z}^{2}}{2} + \cdots. \end{aligned} \right \} $$

Using these values and from (6.3) it follows that

6.16 $$ \left . \begin{aligned} g(z,\bar{z}) & = \bar{v}^{*}(0)f_{0}(z,\bar{z}) \\ & = \bar{v}^{*}(0)f_{0}(z,X_{t}) \\ & = \Delta _{k}\bar{D} \begin{bmatrix} 1, & \bar{v_{1}}^{*}, & \bar{v_{2}}^{*} \end{bmatrix} \begin{bmatrix} X_{2t}(-1)X_{3t}(-1)-X_{2t}(-1)X_{3t}^{2}(-1) \\ 0 \\ - X_{2t}(-1)X_{3t}(-1) \end{bmatrix} \\ & = \Delta _{k}\bar{D} \bigl[X_{2t}(-1)X_{3t}(-1)-X_{2t}(-1)X_{3t}^{2}(-1)-X_{2t}(-1)X_{3t}(-1) \bar{v_{2}}^{*} \bigr]. \end{aligned} \right \} $$

Putting the values of $X_{2t}(-1)$, $X_{3t}(-1)$, and $X_{3t}^{2}(-1)$, computing the above expressions (6.16), and comparing the coefficients of $z^{2}$, *zz̄*, $\bar{z}^{2}$, and $z^{2}\bar{z}$ with (6.15), we have

6.17 $$ \left . \begin{aligned} &g_{20} = 2 \Delta _{k}\bar{D} \bigl(v_{1}v_{2}e^{-2\iota \omega _{0} \Delta _{k}}- v_{1}v_{2}\bar{v}_{2}^{*}e^{-2\iota \omega _{0}\Delta _{k}} \bigr), \\ &g_{11} = \Delta _{k}\bar{D} \bigl(v_{1} \bar{v}_{2}+\bar{v}_{1}v_{2}-v_{1} \bar{v}_{2}\bar{v}_{2}^{*}- \bar{v}_{1}v_{2}\bar{v}_{2}^{*} \bigr), \\ &g_{02} = 2\Delta _{k}\bar{D} \bigl( \bar{v}_{1}\bar{v}_{2}e^{2\iota \omega _{0}\Delta _{k}} - \bar{v}_{1}\bar{v}_{2}\bar{v}_{2}^{*}e^{2 \iota \omega _{0}\Delta _{k}} \bigr), \\ &g_{21} = 2\Delta _{k}\bar{D} \biggl[v_{1}e^{-\iota \omega _{0}\Delta _{k}}W_{11}^{3}(-1) + \frac{\bar{v}_{2}}{2}e^{\iota \omega _{0}\Delta _{k}}W_{20}^{2}(-1) + v_{2}e^{-\iota \omega _{0}\Delta _{k}}W_{11}^{2}(-1) \\ &\hphantom{g_{21} ={}}{} +\frac{\bar{v}_{1}}{2}e^{\iota \omega _{0}\Delta _{k}}W_{20}^{3}(-1)- 2v_{1} \vert v_{2} \vert ^{2}e^{-\iota \omega _{0}\Delta _{k}}- \bar{v}_{1}v_{2}^{2}e^{- \iota \omega _{0}\Delta _{k}} \\ &\hphantom{g_{21} ={}}{} - \bar{v}_{2}^{*} \biggl\{ v_{1}e^{-\iota \omega _{0}\Delta _{k}}W_{11}^{3}(-1)+ \frac{\bar{v}_{2}}{2}e^{\iota \omega _{0}\Delta _{k}}W_{20}^{2}(-1) + v_{2}e^{-\iota \omega _{0}\Delta _{k}}W_{11}^{2}(-1) \\ &\hphantom{g_{21} ={}}{}+ \frac{\bar{v}_{1}}{2}e^{\iota \omega _{0}\Delta _{k}}W_{20}^{3}(-1) \biggr\} \biggr], \end{aligned} \right \} $$

and

6.18 $$ \left . \begin{aligned}& W_{20}(\theta ) = \frac{\iota g_{20}}{\omega _{0}\Delta _{k}}q(0)e^{ \iota \omega _{0}\Delta _{k}\theta }+ \frac{\iota \bar{g}_{02}}{3\omega _{0}\Delta _{k}} \bar{q}(0)e^{- \iota \omega _{0}\Delta _{k}\theta }+ E_{1}e^{2\iota \omega _{0} \Delta _{k}\theta }, \\ &W_{11}(\theta ) = -\frac{\iota g_{11}}{\omega _{0}\Delta _{k}}q(0)e^{ \iota \omega _{0}\Delta _{k}\theta }+ \frac{\iota \bar{g}_{11}}{\omega _{0}\Delta _{k}}\bar{q}(0)e^{-\iota \omega _{0}\Delta _{k}\theta }+E_{2}, \end{aligned} \right \} $$

where $E_{1} = (E_{1}^{(1)},E_{1}^{(2)},E_{1}^{(3)})$ and $E_{2} = (E_{2}^{(1)}, E_{2}^{(2)}, E_{2}^{(3)})$ are a constant vector in $\mathbb{R}^{3}$ satisfying the following equations:

6.19 $$\begin{aligned}& \begin{pmatrix} -1-\iota \omega _{0} & \frac{\bar{z}}{1+\bar{z}}e^{-\iota \omega _{0} \Delta _{k}} & \frac{\bar{y}}{(1+\bar{z})^{2}}e^{-\iota \omega _{0} \Delta _{k}} \\ a_{1} & -a_{2}-\iota \omega _{0} & 0 \\ 0 & -\bar{z}e^{-\iota \omega _{0}\Delta _{k}} & a_{4}-\iota \omega _{0}- \bar{y}e^{-\iota \omega _{0}\Delta _{k}} \end{pmatrix} \begin{pmatrix} E_{1}^{(1)} \\ E_{1}^{(2)} \\ E_{1}^{(3)} \end{pmatrix} = 2 \begin{pmatrix} \Gamma _{11} \\ \Gamma _{21} \\ \Gamma _{31} \end{pmatrix} , \end{aligned}$$

6.20 $$\begin{aligned}& \begin{pmatrix} -1& \frac{\bar{z}}{1+\bar{z}}& \frac{\bar{y}}{(1+\bar{z})^{2}} \\ a_{1} & -a_{2} & 0 \\ 0 & -\bar{z}& a_{4}-\bar{y} \end{pmatrix} \begin{pmatrix} E_{2}^{(1)} \\ E_{2}^{(2)} \\ E_{2}^{(3)} \end{pmatrix} = 2 \begin{pmatrix} \Gamma _{12} \\ \Gamma _{22} \\ \Gamma _{32} \end{pmatrix} , \end{aligned}$$

6.21 $$\begin{aligned}& \left . \begin{aligned} &\Gamma _{11} = v_{1}v_{2}e^{-2\iota \omega _{0}\Delta _{k}}, \\ &\Gamma _{12} = v_{1}\bar{v}_{2}+ \bar{v}_{1}v_{2}, \\ &\Gamma _{21} = 0, \\ &\Gamma _{22} = 0, \\ &\Gamma _{31} = -v_{1}v_{2}e^{-2\iota \omega _{0}\Delta _{k}}, \\ &\Gamma _{32} = -v_{1}\bar{v}_{2}- \bar{v}_{1}v_{2}. \end{aligned} \right \} \end{aligned}$$

Furthermore, we can compute $g_{21}$ with respect to parameters and delay. Hence, from the above analysis we can conclude that in order to find each $g_{ij}$ we have to use the parameters and delay in system (2.2). Thus we can compute the following values:

6.22 $$ \left . \begin{aligned} &c_{1}(0) = \frac{\iota }{2\Delta _{k}\omega _{0}} \biggl(g_{11}g_{20}-2 \vert g_{11} \vert ^{2}- \frac{ \vert g_{02} \vert ^{2}}{3} \biggr)+ \frac{g_{21}}{2}, \\ &\mu _{2} = - \frac{\operatorname{Re}\{c_{1}(0)\}}{\operatorname{Re}\{\lambda ^{\prime }(\Delta _{k})\}}, \\ &\beta _{2} = 2\operatorname{Re} \bigl\{ c_{1}(0) \bigr\} , \\ &T_{2} = - \frac{\operatorname{Im}\{c_{1}(0)\}+\mu _{2}\operatorname{Im}\lambda ^{\prime }(\Delta _{k})}{\Delta _{k}\omega _{0}}. \end{aligned} \right \} $$

Based on our analysis, by the result of Hassard *et al.* [35], we have the following result.

### Theorem 6.1

*The sign of* $\mu _{2}$, $\beta _{2}$, *and* $T_{2}$ *determines the directions of Hopf bifurcations*, *stability of the bifurcating periodic solutions*, *and the period of bifurcating periodic solutions respectively for* $\Delta = \Delta _{k}$. *In view of* (6.22), *the following results hold for system* (2.2):

- *If* $\mu _{2} < 0$ ($\mu _{2} > 0$), *the Hopf bifurcation is subcritical* (*supercritical*).
- *If* $\beta _{2} > 0$ ($\beta _{2} < 0$), *the bifurcation periodic solutions are unstable* (*stable*).
- *If* $T_{2} < 0$ ($T_{2}> 0$), *the period of the bifurcated periodic solution decreases* (*increases*).

## Numerical simulations

**Figure 1:**
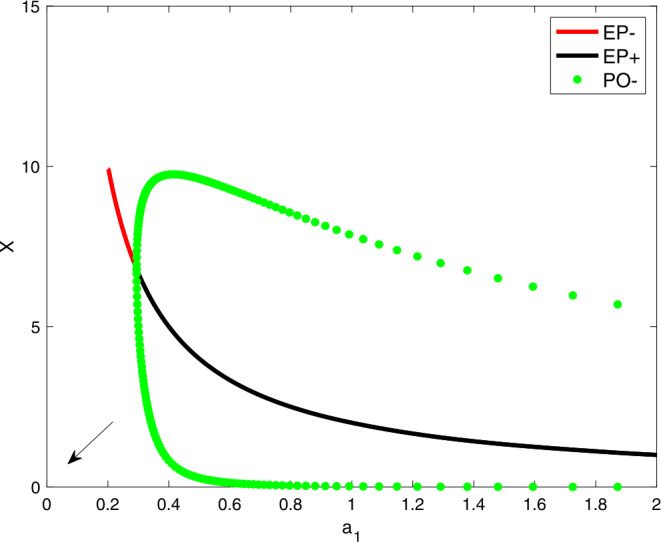
A bifurcation diagram showing *X* as a function of $a_{1}$. Parameter values are $a_{1}=0.3$, $a_{2}=0.6$, $a_{3}=4$, $a_{4}=10$, and $\Delta =0.05$

Through our above analysis, we have gained an analytical understanding of the possible dynamics of our proposed nonlinear delay differential equation model (2.2). In this section, bifurcation analysis and parameter sensitivity will be discussed. The delay model (2.2) showed that the system exhibited oscillations. Here, we shall discuss the effects of varying the parameter $a_{1}$ on generating the oscillatory behavior. In Fig. 1, *X* is written as a function of $a_{1}$. The curves (equilibrium branch) having red and black colors represent the stable and unstable steady state branches, respectively. Further, the green curve denotes the maximum and minimum of the limit cycle. The equilibrium branch loses its stability due to the appearance of a Hopf bifurcation point. In the unstable branch, the system (2.2) exhibits oscillations, and from the figure, it can be observed that the amplitude of these oscillations decreases as $a_{1}$ increases.

**Figure 2:**
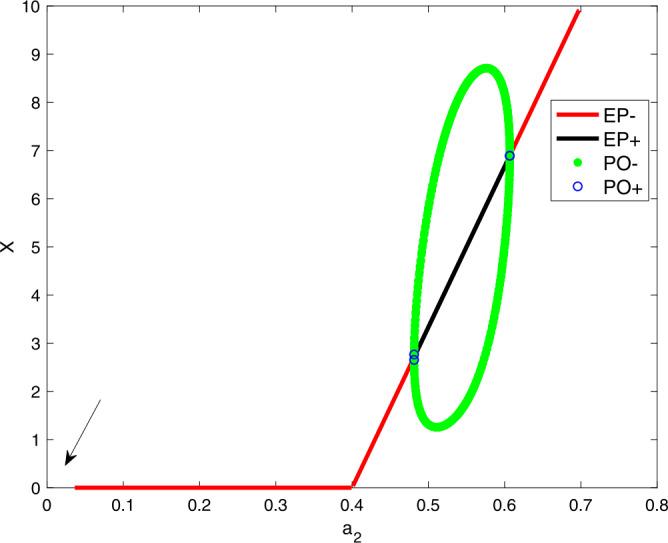
A bifurcation diagram showing *X* as a function of $a_{2}$. Parameter values are $a_{1}=0.3$, $a_{2}=0.6$, $a_{3}=4$, $a_{4}=10$, and $\Delta =0.05$

Similar effects of $a_{2}$ on the steady states of *X* are discussed in Fig. 2. The red, black curves represent the stable and unstable steady state branches, whereas the green circle denotes the stable limit cycles. For low and high values of $a_{2}$, the system (2.2) has a stable equilibrium point. The system (2.2), however, does not converge to a steady-state when $a_{1}=0$ or $a_{2}=0$. These are shown by arrows in Figs. 1 and 2. The reason for this is because the equilibrium expression $E_{1}$ is divided by $a_{2}$ and $E_{2}$ is divided by $a_{1}$. Hence, $E_{1}, E_{2} \rightarrow \infty $.

After discussing the effects of $a_{1}$ and $a_{2}$ on generating limit cycle oscillations, we need to investigate how the interplay between the two parameters $a_{1}$ and $a_{2}$ can alter these oscillations. To address this question, we shall track the two parameters on a two-parameter plot.

**Figure 3:**
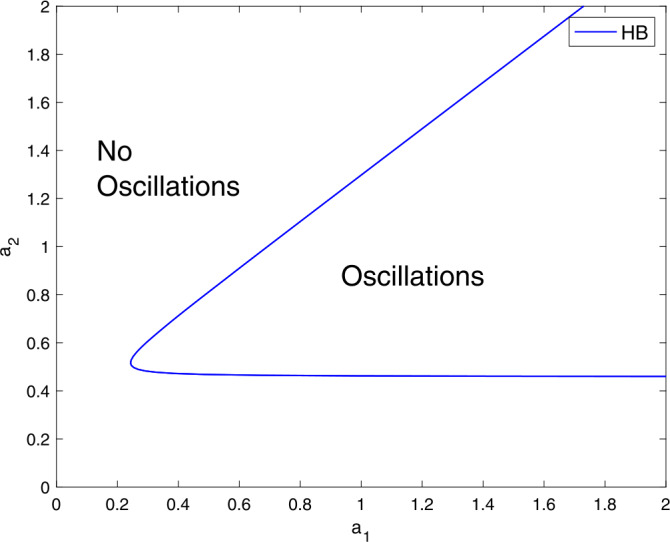
A two-parameter plot $a_{1}$ vs $a_{2}$ showing the effects of these two parameters on the oscillations. Parameter values are similar to Fig. 1

Figure 3 shows a two-parameter plot. Within the cusp, the system (2.2) exhibits oscillations. Outside the cusp, the model (2.2) only has a single stable steady-state. In the absence of $a_{1}$ or $a_{2}$, the system cannot generate limit cycle oscillations. For the model (2.2) to generate an oscillatory response, it must have a “well” balance between $a_{1}$ and $a_{2}$. If this balance is biased, the oscillatory response cannot be achieved.

**Figure 4:**
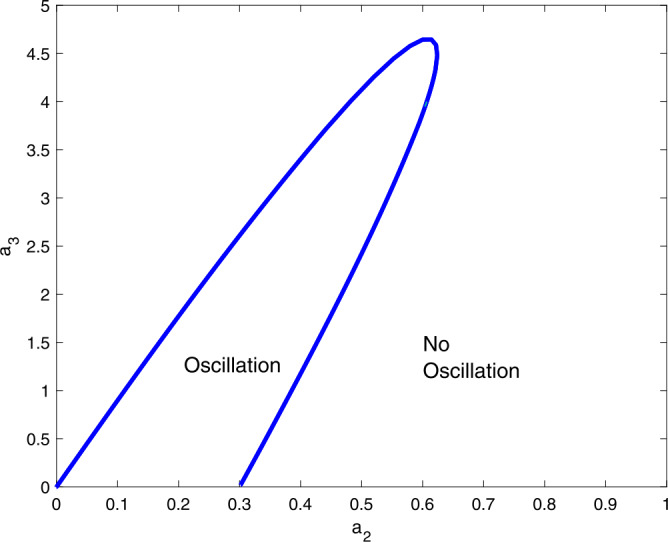
A two-parameter plot $a_{2}$ vs $a_{3}$ showing the effects of these two parameters on the oscillations. Parameter values are similar to Fig. 1

When selecting values of $a_{1}$ and $a_{2}$ from the region of oscillations of the figure, the model (2.2) shows oscillation. We shall focus on the effects of $a_{3}$ on these oscillations. In other words, how does it alter this response? Figure 4 shows a two-parameter bifurcation diagram, and it shows that if either of these two parameters increases beyond a critical level, the oscillations will be destroyed. Additionally, in the absence of $a_{3}$, the model (2.2) can generate an oscillatory behavior.

**Figure 5:**
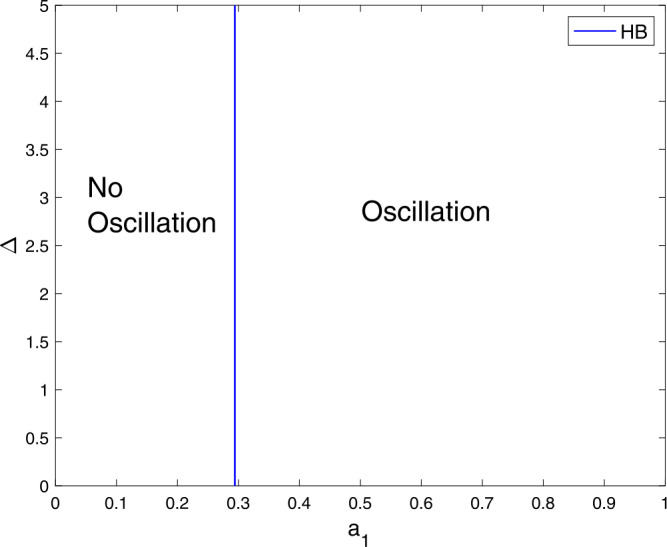
The interplay between $a_{1}$ and Δ in order to generate oscillations. Parameter values are similar to Fig. 1

Here, we shall focus numerically on the effects of the delay term on the limit cycle oscillations. Figure 5 illustrates the relationship between $a_{1}$ and Δ on generating limit cycle oscillations. In this Figure, in the absence of the delay term, the system (2.2) can generate oscillations if $a_{1}$ is increased beyond the blue vertical line (the Hopf locus).

**Figure 6:**
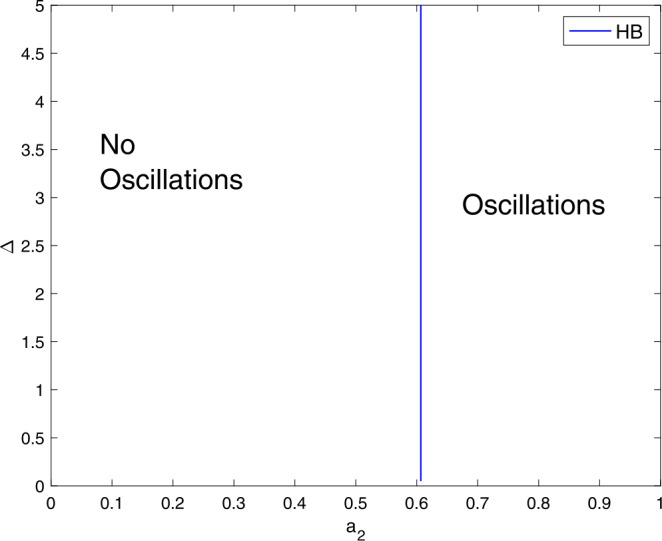
A two-parameter plot showing the role of $a_{2}$ and Δ on generating limit cycle oscillations. Parameter values are similar to Fig. 1

We shall consider the role of $a_{2}$ and the delay term on generating oscillations and this can observe in Fig. 6. The system (2.2) can generate an oscillatory behavior even without the delay term as shown in Pang *et al.* model [17]. Figure 5 and Fig. 6 suggests that the system (2.2) requires greater $a_{2}$ value than $a_{1}$ value in order to be able to generate oscillations.

**Figure 7:**
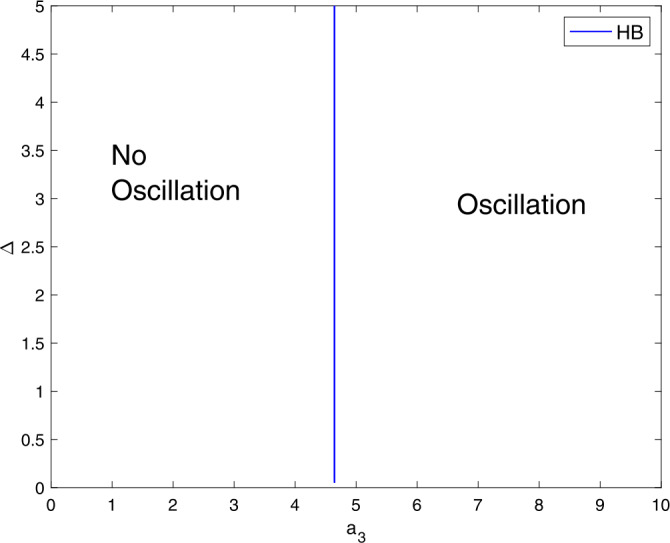
A two-parameter plot showing the role of $a_{3}$ and Δ on generating limit cycle oscillations. Parameter values are similar to Fig. 1

Figure 7 highlights the contribution of the parameters $a_{3}$ and Δ on destabilizing the stable steady state branch. For the model (2.2) to generate oscillations, it requires greater $a_{3}$ values than $a_{1}$ and $a_{2}$. Thus, the system (2.2) can cross the Hopf locus and generate an oscillatory behavior.

**Figure 8:**
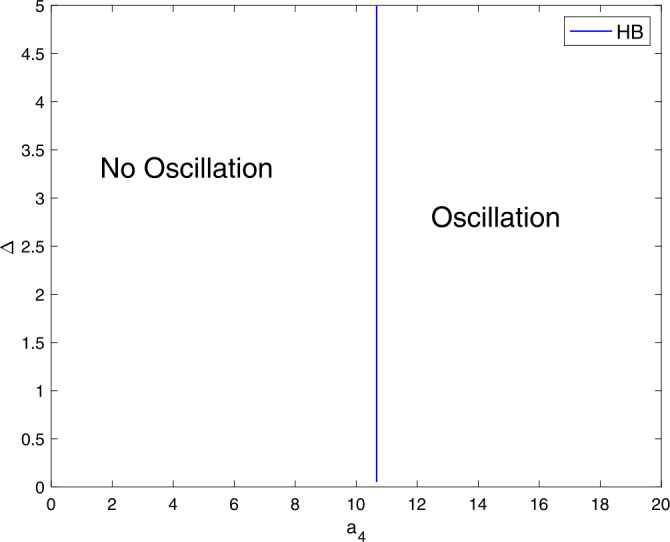
A two-parameter plot showing the role of $a_{4}$ and Δ on generating limit cycle oscillations. Parameter values are similar to those in Fig. 1

Figure 8 shows that the model (2.2) requires greater $a_{4}$ values in order to exhibit oscillations that $a_{1}$, $a_{2}$, and $a_{3}$.

However, suitable parameter values often give a meaningful biological scenario of the system (2.2). Therefore, we perform some simulation works for a better understanding of our analytical treatment. We consider different values of the parameters and the delay factor (Δ) to observe biologically plausible different dynamical scenarios of the model (2.2), enough to merit the mathematical study.

**Figure 9:**
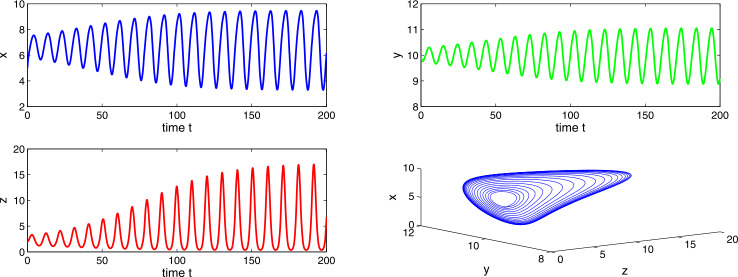
Time evaluation curve and three-dimensional phase portrait of the system for the parameter values $a_{1}=0.3$, $a_{2}=0.6$, $a_{3}=4.0$, $a_{4}=10.0$, $\Delta =0.01$

**Figure 10:**
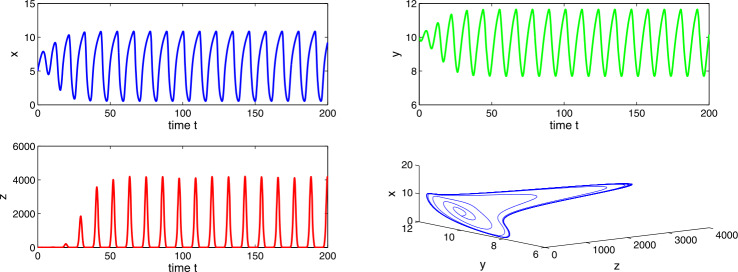
Time evaluation curve and three-dimensional phase portrait of the system for the parameter values $a_{1}=0.3$, $a_{2}=0.6$, $a_{3}=4.0$, $a_{4}=10.0$, $\Delta =0.05$

Choosing $a_{1} = 0.3$, $a_{2}=0.6$, $a_{3}=4.0$, $a_{4}=10.0$, then $a_{2} > a_{1}$ and $(a_{2}-a_{1})a_{4}< a_{3}< a_{2}a_{4}$, which implies the existence of equilibria $E_{1}$ and $E_{2}$. Using the conditions of local stability, we get that both the equilibria $E_{1}$ and $E_{2}$ are unstable. Also, there occurs a periodic solution at $E_{2}$. Figures 9 and 10 show the oscillating behavior as well as the periodic solutions for the system (2.2). Existence of periodic solutions is relevant in cancer models. It implies that the tumor levels may oscillate around a fixed point even in absence of any treatment. Such a phenomenon, which is known as Jeff’s phenomenon, has been observed clinically. We observe that Δ is beneficial for tumor cells. We observe no stability switch in the system (2.2) as the delay factor Δ increases.

**Figure 11:**
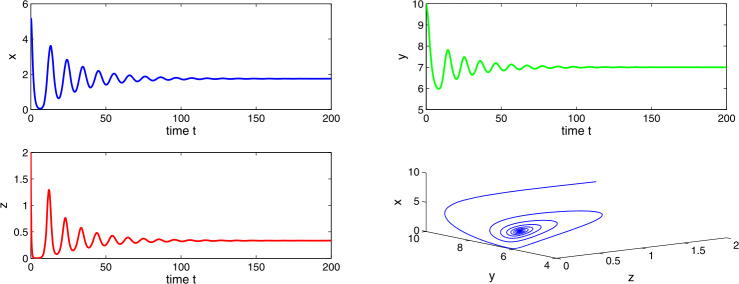
Time evaluation curve and three-dimensional phase portrait of the system for the parameter values $a_{1}=0.4$, $a_{2}=0.6$, $a_{3}=3.5$, $a_{4}=7.0$, $\Delta =0.01$

**Figure 12:**
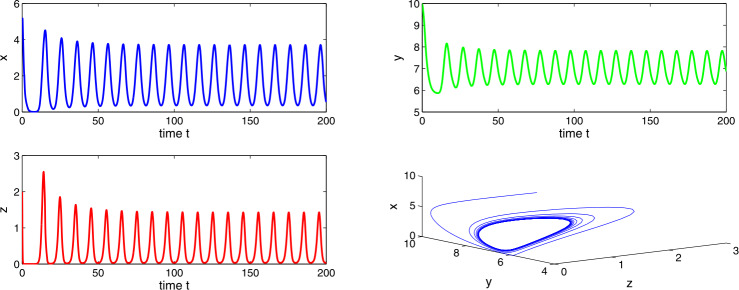
Time evaluation curve and three-dimensional phase portrait of the system for the parameter values $a_{1}=0.4$, $a_{2}=0.6$, $a_{3}=3.5$, $a_{4}=7.0$, $\Delta =0.05$

Choosing $a_{1} = 0.4$, $a_{2}=0.6$, $a_{3}=3.5$, $a_{4}=7.0$, then $a_{2} > a_{1}$ and $(a_{2}-a_{1})a_{4}< a_{3}< a_{2}a_{4}$, which implies the existence of equilibria $E_{1}$ and $E_{2}$. Using the conditions of local stability, we get tumor-free equilibrium $E_{1}$ as unstable and co-axial equilibrium $E_{2}$ as stable in nature. From Figs. 11 and 12, we observe a stability switch in the system (2.2) as the delay factor Δ crosses a threshold.

**Figure 13:**
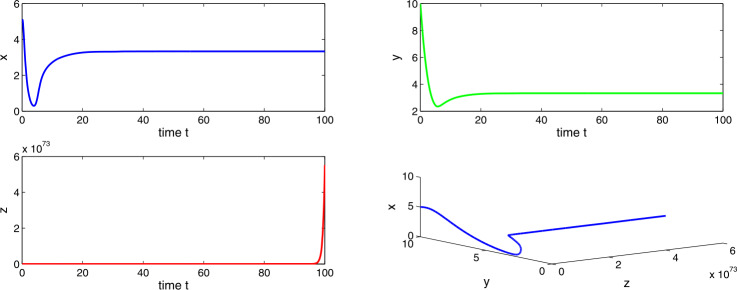
Time evaluation curve and three-dimensional phase portrait of the system for the parameter values $a_{1}=0.3$, $a_{2}=0.6$, $a_{3}=1.0$, $a_{4}=5.0$, $\Delta =0.01$

**Figure 14:**
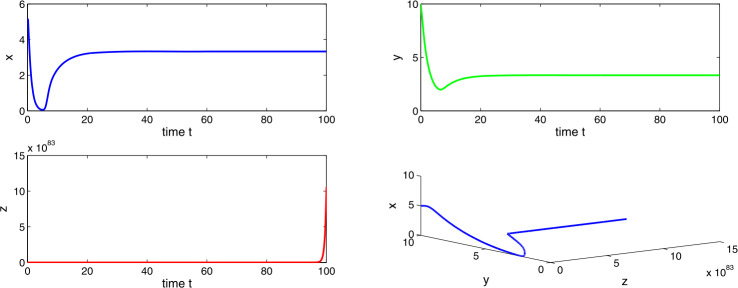
Time evaluation curve and three-dimensional phase portrait of the system for the parameter values $a_{1}=0.3$, $a_{2}=0.6$, $a_{3}=1.0$, $a_{4}=5.0$, $\Delta =0.05$

Choosing $a_{1} = 0.4$, $a_{2}=0.6$, $a_{3}=3.5$, $a_{4}=7.0$, then $a_{2} > a_{1}$ and $0 < a_{3} <(a_{2}-a_{1})a_{4}$, which indicates the existence of tumor-free equilibrium $E_{1}$. At this equilibrium the system (2.2) shows unstable behavior. Figures 13 and 14 indicate that the number of tumor cells increases without restriction, which is in accordance with the immune escape phenomena of tumor which is observed clinically.

**Figure 15:**
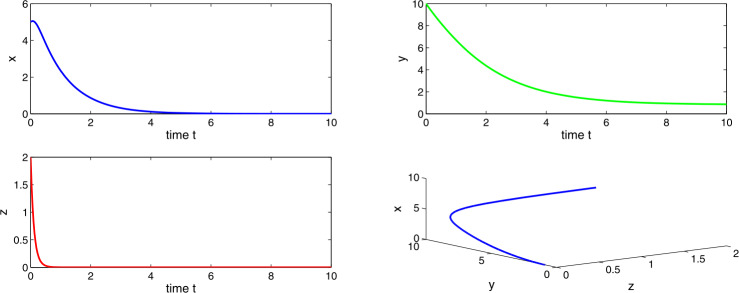
Time evaluation curve and three-dimensional phase portrait of the system for the parameter values $a_{1}=0.3$, $a_{2}=0.6$, $a_{3}=0.5$, $a_{4}=0.7$, $\Delta =0.01$

**Figure 16:**
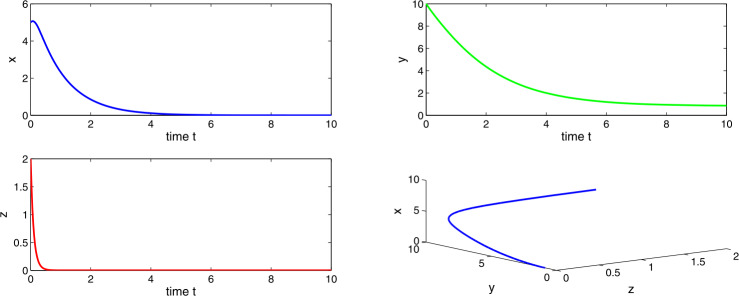
Time evaluation curve and three-dimensional phase portrait of the system for the parameter values $a_{1}=0.3$, $a_{2}=0.6$, $a_{3}=0.5$, $a_{4}=0.7$, $\Delta =0.05$

Choosing $a_{1} = 0.3$, $a_{2}=0.6$, $a_{3}=0.5$, $a_{4}=0.7$, then $a_{3} > a_{2}a_{4}$, which suggests the existence of the stable tumor-free equilibrium $E_{1}$. Figures 15 and 16 clarify that the tumor-free equilibrium is asymptotically stable, which is in accordance with the spontaneous tumor regression phenomena observed clinically.

## Conclusion

In this paper, we aimed to investigate the dynamical behavior of the modified Pang *et al.* model [17]. In their model [17], it was shown that with the increase of normal flow rate of mature immune cells, the system exhibits different states such as tumor dormant, periodic tumor oscillation, immune escape of tumor, and so on. However, the effects of the delay term on the oscillatory behavior were not considered in their model. Therefore, a delay term was included in our model, and we investigated the system behavior with varying system parameters. As a result, the modified model showed that the system (2.2) could generate an oscillatory response even with a delay term. Moreover, it illustrated that these oscillations were persistent and could not be destroyed by the additional delay term. Our bifurcation analysis and numerical simulations revealed that a “careful” selection of the model’s parameters must be obtained so that the stable steady-state loses its stability. We showed that the delay term was not necessary to generate oscillations because our model can generate these oscillations even without the delay term. From the bifurcation analysis, in addition to Figs. 9 and 10, it can be shown that Δ neither affects generating of oscillations nor the amplitude of these oscillations. However, varying other parameters such as but not limited to $a_{1}$, $a_{2}$ leads to the stabilization of the unstable equilibrium point. A set of realistic parameter values gives us a better insight into the model, which we leave as our future work.

In our entire discussion, the major goal was to have a dynamical analysis of the considered model with the incorporation of a delay term. The numerical results were obtained by applying standard MATLAB software. The aspects of numerical stability, CPU time, minimum error, etc. of the adopted numerical techniques were not investigated. The interested readers and researchers are referred to [36, 37] for such kinds of investigations.

## Data Availability

Not applicable.
